# A model of hematopoietic bone marrow apoptosis during growth factor deprivation in combination with a cytokine

**DOI:** 10.1186/s12976-018-0080-2

**Published:** 2018-06-21

**Authors:** Christina L. Mouser, Eliana S. Antoniou, Evros K. Vassiliou

**Affiliations:** 1 Department of Mathematics, William Paterson University, 300 Pompton Rd, Wayne, NJ 07470 USA; 2 School of Natural Sciences, Kean University, 1000 Morris Ave, Union, NJ 07083 USA

**Keywords:** Hematopoiesis, Growth factor deprivation, Differential delay equations

## Abstract

**Background:**

The process by which blood cells are formed is referred to as hematopoiesis**.** This process involves a complex sequence of phases that blood cells must complete. During hematopoiesis, a small fraction of cells undergo cell death. Causes of cell death are dependent upon various factors; one such factor being growth factor deprivation.

**Methods:**

In this paper, a mathematical model of hematopoiesis during growth factor deprivation is presented. The model consists of a set of three coupled differential delay equations. Phase plane and linear stability analysis are performed in order to locate and determine stability of fixed points. Numerical simulations of the governing equations are run and provide a visual display of the behavior of the stem cell population undergoing growth factor deprivation. In addition, the effect of cytokine administration is incorporated in the model in an effort to understand how cytokine administration can offset the negative effects of apoptosis caused by growth factor deprivation.

**Conclusions:**

The model produces qualitatively similar results to that observed during serum deprivation. The model captures apoptosis levels of cells at different time points. Additionally, it is shown that cytokine administration stabilizes the stem cell count.

## Background

Hematopoiesis is the biological process by which hematopoietic stem cells (HSC) located in the bone marrow generate blood cells [1]. HSCs are responsible for the daily production of blood cells estimated to be approximately 10^11^–10^12^ in a healthy individual [2]. Understanding the exact process that occurs during the cell cycle is a significant problem because there are several diseases associated with inadequate production and maintenance of blood cells. Multicellular organism development relies on a delicate balance between cell proliferation and cell death (apoptosis) [3]. While cell proliferation results in the development of various tissues and organs, cellular death leads to refinement of the various tissues and ultimately produces functional organs. Imbalances in cell proliferation and cell death result in a variety of diseases ranging from neurodegeneration to tumorigenesis [4, 5]. Cells undergoing serum (blood plasma not including fibrinogens) deprivation exhibit signs of apoptosis [6]. In some cases, cells subject to cytokines may show signs of proliferation [7]. In other cases, cytokines may exert a negative feedback on hematopoietic mechanisms in conditions of high cell counts in circulation [8]. As such, cytokines act as regulatory proteins that are released by cells of the immune system and control the self-renewing process of stem cells [9]. There are numerous types of cytokines some of which are erythropoietin (EPO), granulocyte colony-stimulating factor (G-CSF), and Interleukin-1 through Interleukin-12. The cytokine EPO, for example, has been shown to intensify the production of red blood cells in response to oxygen levels in the body [10]. Interleukin-3 has been shown to enhance the survival of HSCs while G-CSF has been shown to contribute to mobilization of HSCs [11–13]. Based on previously published work, we develop a mathematical model that captures the effect of growth factor deprivation and cytokine administration [14, 15]. Our goal is to model the phenomenon observed in the presence of cytokines offsetting the negative effects of growth factor deprivation.

The model presented in this paper captures the behavior observed during serum deprivation. Based on flow cytometric studies conducted by others, the set of coupled differential delay equations captures apoptosis measured as PS plasma translocation levels of cells at different time points [16–18].

Additionally, we incorporate the effect of cytokines in our model. Cytokines have experimentally been observed to trigger an increase in proliferation of quiescent human stem cells [19]. Therefore, it is important to include a mathematical term to account for the effect of cytokines in the model equations in an effort to determine if cytokines can stabilize cell count. In order to better understand the dynamics of the model, phase plane analysis is carried out. Linear stability analysis is also performed in order to locate and determine stability of fixed points and simulations of the model are run in XPPAUT [20].

## Materials and methods

### The model

Using the model previously constructed and published in [15], we expand to include the effect of growth factor deprivation on a population of proliferating and non-proliferating cells. This model describes the number of proliferating and non-proliferating stem cells by a set of coupled delay differential equations. As opposed to our previous work, we have assumed a fixed oxygen concentration in the model. The modeling set of equations is:

1 $$ \frac{dP}{dt}=-\gamma P+\beta (N)N-{\exp}^{-\gamma \tau}\beta \left({N}_{\tau}\right){N}_{\tau }+{\beta}_c\left({P}_{\tau}\right){N}_{\tau }-\overline{\mathrm{r}}(s)\  rP,\kern1.56em \tau <t $$

2 $$ \frac{dN}{dt}=-\left(\beta (N)N+\delta N\right)+2{\exp}^{-\gamma \tau}\beta \left({N}_{\tau}\right){N}_{\tau }-{\beta}_c(P)N,\kern5.5em \tau <\mathrm{t} $$

3 $$ \frac{dA}{dt}=\overline{\mathrm{r}}(s)\  rP+\gamma P $$

4 $$ \beta (N)={\beta}_0\frac{\theta^n}{\theta^n+{N}^n} $$

5 $$ {\beta}_c(P)={\beta}_{0,c}\frac{\theta_1^m}{\theta_1^m+{P}^m}{g}_c(t) $$

6 $$ {g}_c(t)=\left\{\begin{array}{cc}1,& 0<t\le {\tau}_1\\ {}{\mathit{\exp}}^{-{s}_1\left(t-{\tau}_1\right)},& t>{\tau}_1\end{array}\right. $$

In the above equations, *P* represents the number of proliferating stem cells and *N*represents the number of non-proliferating cells. *β*(*N*) (eq. (4)) measures the rate of cell re-entry into proliferation, *β*_0_ is the maximal rate of cell transit from resting phase to S phase, n measures the sensitivity of the rate of cell transit from G0 phase to S phase, and *θ* is the G0 stem cell population at which the rate of cell movement from G0 into proliferation is one-half of its maximal value. *δ* is the rate of random cell loss by escape to the periphery and *γ* is the rate of cell loss due to apoptosis. *τ* is the time required for a cell to complete one cycle of the proliferation phase. The notation *N*_*τ*_, for example, represents *N*(*t* − *τ*), thus introducing a time delay into the equations. The values of parameters stated above are based on estimates given by in [21–25].

Growth factor deprivation contributes to cell death and, therefore, we subtract $\overline{r}(s) rP$ in eq. (1). We define $\overline{r}(s)$ as a maximal rate of cell death as a function of growth factor deprivation, *s*, and we define *r* as a uniformly distributed random variable from the interval (0, 0.0005) so that the variability of the effect of growth factor deprivation is within a .05% interval. We keep track of the number of apoptotic cells by introducing the variable *A* whose rate of change is given by eq. (3). Note that cell death can be attributed to both growth factor deprivation and apoptosis and determined through the parameter *γ*.

The term *β*_*c*_(*P*)*N* in eqs. (1) and (2) models the effect of cytokines on the proliferating and non-proliferating stem cells and is a Hill function. This term models the effect of cytokines by creating a cell loss of the non-proliferating population and a cell gain of the proliferating population when the proliferating cell population is low. This represents the transition of non-proliferating to proliferating cells as a result of cytokine administration. Since the effects of cytokines decay with time, we multiply by *g*_*c*_(*t*) which causes the effect of cytokine administration to exponentially decay. The parameter *s*_1_determines the rate of decay of the cytokines and *τ*_1_is a parameter that sets the time at which the effect of cytokines begins to decay. The parameter values in the term *β*_*c*_(*P*) were chosen to be consistent with realistic time frames of cytokines as noted in [26].

### Steady-state solutions of the model and stability analysis

Steady-state solutions for the proliferating and non-proliferating cells have been calculated and linear stability analysis of eqs. (1) and (2) has been carried out as in [14, 15]. Two sets of steady-state solutions were found. The first set is *P*^∗^ = *N*^∗^ = 0. The second and more interesting steady-state solution is:

7 $$ {N}^{\ast }=\theta {\left[\frac{\beta_0\left(2{\exp}^{-\gamma \tau}-1\right)}{\delta +{\beta}_{0,c}}-1\right]}^{1/n} $$

8 $$ {P}^{\ast }=\frac{N^{\ast }}{\Big(\gamma -\overline{r}(s)r}\left[{\beta}_{0,c}+\left(\delta +{\beta}_{0,c}\right)\frac{\left(1-{\exp}^{-\gamma \tau}\right)}{\left(2{\exp}^{-\gamma \tau}-1\right)}\right] $$

The nontrivial steady state exists only if

9 $$ 0<\gamma \tau \kern0.5em <\ln \frac{2{\beta}_0}{\delta +{\beta}_{0,c}+{\beta}_0}<\ln 2 $$

Also, $$ \gamma =-\overline{r}(s)r $$ and $$ \frac{\delta +{\beta}_{0,c}}{\gamma -\overline{r}(s)r}>0 $$.

Stable solutions of eqs. (1) and (2) exist only under the condition:

$\omega \tau <{\cos}^{-1}\left(\frac{-A}{B}\right)$, where *A* = *δ* + *β*_0_*F* + *β*_0, *c*_, *B* =  − 2*β*_0_exp^−*γτ*^*F* and $F={\theta}^n\left[\frac{\theta +{N}^{\ast n}\left(1-n\right)}{{\left({\theta}^n+{N}^{\ast n}\right)}^2}\right]$.

With the restriction that $\left|\frac{A}{B}\right|<1.$.

Biologically, *γ* captures the apoptosis rate which has to be within a certain range for the system to remain active. The condition in eq. (9) reflects the fact that if apoptosis exceeds certain values, hematopoiesis will cease leading to death. Lastly, for homeostasis to prevail, the collection of parameter values must yield dynamic equilibrium (as given by eqs. (7) and (8)). Parameter values used in our model are given in Table 1 and were found to satisfy the above conditions.

**Table 1:** Parameter values used in simulations of model

*P*_0_	1000 cells
*N*_0_	10,000 cells
*δ*	0.07/days
*γ*	0.1/days
*τ*	2.22 days
*θ*	650
*θ*_1_	450
*β*_0_	16
*β*_0, *C*_	0 (baseline case)
	0.022 (cytokine administration)
*n*	3
*m*	3
*s*_1_	0.01
*r*	0 (baseline case)
	0.07 (growth factor deprivation)
*τ*_1_	100

## Results

**Fig. 1:**
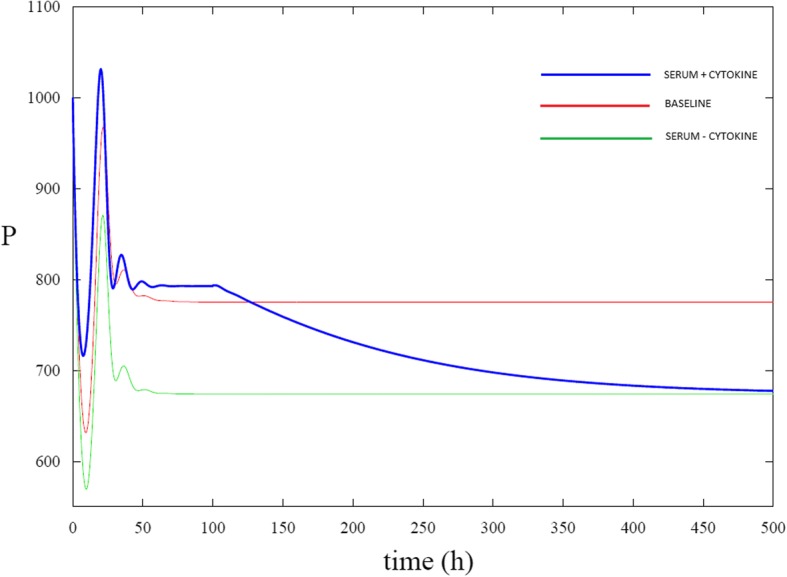
Proliferating stem cell count determined through simulations of the model. The number of proliferating cells (P) are plotted against time. The red curve shows P in the absence of growth factor deprivation and cytokine administration (baseline case). The green curve shows P in the presence of growth factor deprivation. The blue curve shows P with both growth factor deprivation and cytokine administration. By comparing the green and red curves, it can be seen that growth factor deprivation in the absence of cytokine administration causes a sharp decrease in the number of proliferating stem cells. When cytokine is administered in conjunction with serum deprivation (blue curve), the proliferating stem cell count is significantly larger in comparison with the no-cytokine case and closely matches the baseline case towards the beginning of the simulation. As the cytokine wears off with time, the number of proliferating cells becomes closer to that of when serum deprivation is solely present

**Fig. 2:**
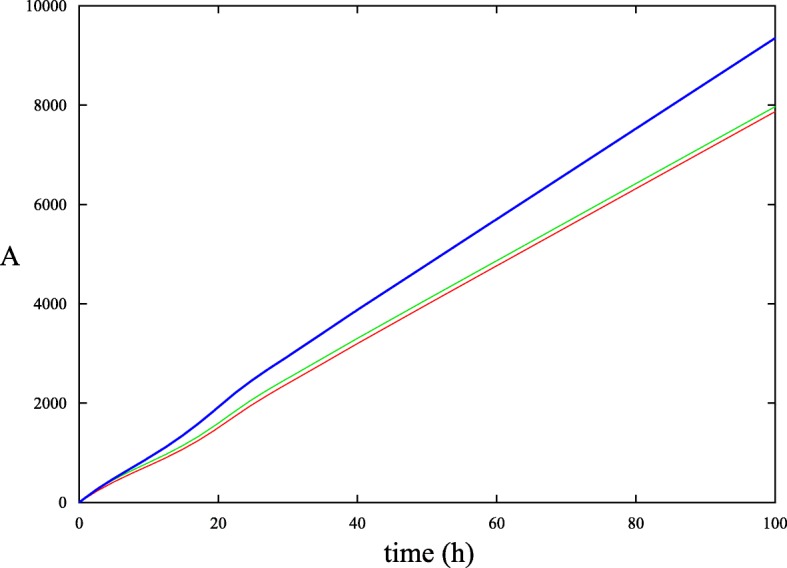
Apoptotic cell count determined through simulations of the model. The number of apoptotic cells (A) is plotted against time. The red curve shows A in the absence of growth factor deprivation and cytokine administration (baseline case). The green curve shows A during the presence of growth factor deprivation. The blue curve shows A during growth factor deprivation and cytokine administration. Note that cytokine administration does not prevent cells from undergoing apoptosis but rather boosts the rate of proliferation of healthy cells

The system of eqs. (1)–(6) were solved numerically using the software XPPAUT under various conditions. See Figs. 1 and 2 below. Simulations were run in the baseline case meaning that growth factor deprivation and cytokine administration were not included in the model. The initial conditions were chosen so that *P*=1000, N=10,000 and *A* = 0. The ratio of *P* vs *N* is consistent with HSC measurements conducted using young adult bone marrow [27]. In this case, the number of proliferating stem cells exhibited damped oscillations and then reached a steady state value of approximately 775 cells. Next, simulations were run with growth factor deprivation included in the model. With growth factor deprivation, the number of proliferating stem cells continued to initially exhibit damped oscillations but at smaller values of the proliferating cell count. As was expected, the number of proliferating cells reached a smaller equilibrium value (approximately 674 cells) than in the baseline case. A comparison of the number of apoptotic cells in the simulations run in the baseline case versus simulations run with growth factor deprivation show that cell death occurred at a higher rate when growth factor deprivation was incorporated in the modeling set of equations. Finally, simulations were run with growth factor deprivation and cytokine administration both included in the model. In this case, cytokine administration was able to counter the adverse effect of growth factor deprivation. Cytokine administration boosts the proliferating cell count. With an appropriate strength (dosage) of the cytokine administration, the number of proliferating stem cells underdoing growth factor deprivation can be brought back to a value that is in close proximity to the baseline case. Fig. 1 shows a comparison of the number of proliferating stem cells in the three cases: baseline, with growth factor deprivation but not cytokine administration, and with both growth factor deprivation and cytokine administration. The number of proliferating cells is initially the same in the simulations. Serum deprivation causes the number of proliferating cells to decrease in comparison to the baseline case and cytokine administration causes the number of proliferating cells to shoot up in comparison to the baseline and serum deprived cases. As the number of proliferating cells settles towards the equilibrium (at approximately t=150 hrs), the baseline case and case with joint growth factor deprivation plus cytokine administration are relatively consistent with one another, while the case solely with growth factor deprivation shows lower numbers of proliferating cells. However, the effect of the cytokine begins to wear off (due to *g*_*c*_(*t*)) causing the number of proliferating cells to decay towards the same steady state value as is reached solely when serum deprivation is present.

In Fig. 2, it can be seen that with growth factor deprivation, the number of apoptotic cells increases as expected. When cytokine in administered in conjunction with growth factor deprivation, the number of proliferating cells greatly increases. This increase in the population of proliferating cells also means that there are more cells available to undergo random cell loss (as determined by *γ*). Thus, in addition to the number of proliferating cells increasing, the number of apoptotic cells also increases in the presence of cytokines.

## Discussion

Based on the simulation results seen in Fig. 1, one can deduce that both cytokine administration and no-cytokine administration will ultimately converge to the same steady levels provided the time period is long enough. This is consistent with homeostatic principles and the overall tendency of a hematopoietic system to restore itself to a normal state. Nonetheless, cytokine administration mobilizes stem cell proliferation in the early stages and results in higher number of proliferating cells in the early stages. Again, this observation is consistent to what is seen in patients who are treated with hematopoietic inducing agents. In similar manner, growth factor deprivation, a scenario typically seen in patients subjected to chemotherapy, there is an increase in the number of proliferating cells. In the first case, this is a result of the presence of proliferating inducing agent and in the latter a result of increased apoptosis due to a chemotherapeutic agent. However, in a scenario of increased apoptosis, the number of proliferating cells is decreased in the long term and unable to reach normal levels. As expected, a low number of proliferating cells at the beginning of the simulation yields a low number of proliferating cells at the end of the simulation.

The number of apoptotic cells is higher in both growth factor deprivation and growth factor deprivation with a cytokine compared with the baseline case, as seen in Fig. 2. The overall effect of the cytokine is to induce a higher number of proliferating cells that compensates for the loss of apoptotic cells. As such, the benefit of using a cytokine is not through the prevention of apoptosis under a stressful event but more to an increase in the number of proliferating cells.

## Conclusion

In conclusion, the model presented exhibits an endpoint where the levels of proliferating stem cells in the presence of cytokine stabilize to a level that is comparable to levels seen in the absence of growth factor deprivation. The theoretical outcome of this model is that cytokines or any inducing hematopoietic agent allow for an increased value of proliferating cells but do not prevent apoptosis. Administration of cytokines during a stressful event, such as chemotherapy, is a practical way of maintaining equilibrium between apoptotic and proliferating.

## Abbreviations

EPO: Erythropoietin
FACS: Flow Cytometry
G-CSF: Granulocyte-Colony Stimulating Factor
HSC: Hematopoietic stem cells
